# Survey the effectiveness of education based on message framing through mobile phone on women’s physical activity

**DOI:** 10.1186/s12889-023-17212-3

**Published:** 2023-11-22

**Authors:** Ghodratollah Shakerinejad, Zahra Baji, Masoumeh Tehrani, Farzaneh Jarvandi, Maria Cheraghi, Nasser Hatamzadeh

**Affiliations:** 1 Health Education Research Department, Academic Center for Education, Culture and Research (ACECR)- Khuzestan, Ahvaz, Iran; 2 Social Determinant of Health Center, Department of Public Health, school of Health, Ahvaz Jundishapur University of Medical Sciences, Ahvaz, Iran; 3 Health Education & Health Promotion Department, school of Health, Ahvaz Jundishapur University of Medical Sciences, Ahvaz, Iran

**Keywords:** Message framing, Physical activity, Female employees, Ahvaz

## Abstract

**Background:**

Physical activity in female employees is a healthy behavior and increases strength, endurance, improves flexibility, improves the feeling of vitality and freshness, improves health, and ultimately increases life expectancy. Health messages are one of the most effective ways to engage people and motivate them to perform healthy behaviors. The purpose of this study was to the study of the effectiveness of education based on message framing through mobile phone (whatsapp) on the physical activity of women employees of universities and higher education institutions in Ahvaz city.

**Method:**

In this interventional study, 90 of female employees of three universities and higher education institutions of Ahvaz city were selected by random sampling and randomly divided into three groups (30 participants) receiving gain framed messages, receiving loss framed messages and the control group. The tools of data collection were demographic information questionnaire and international physical activity questionnaire (IPAQ). The participants of the intervention groups received educational messages about physical activity behavior in two different gain and loss framed messages through whatsapp for one month. Data were collected from three groups at the beginning of the study, immediately and two months after the intervention, and were analyzed using SPSS version 26.

**Results:**

The results showed that there was a significant increase in the average physical activity score after the intervention in two interventional groups. by comparing the increase of this score, 53% improvement in physical activity is observed in the gain message group and 15% in loss massage group but there was no significant increase in the control group.

**Conclusion:**

The results of this study showed that the design and implementation of education programs based on message framing, especially gain framed messages through online education (Whatsapp) can improve and promote physical activity behavior in women employees.

## Introduction

Women’s health is one of the priorities of every society. Therefore, promoting and ensuring women’s health is one of the important pillars of the progress of societies and should always be paid attention to [1]. Regular physical activity can improve women’s health and prevent diseases and conditions that cause death and disability of women all over the world. Physical activity improves the mental health of women by reducing the levels of stress, anxiety, depression and increasing self-esteem. Also, sufficient and regular physical activity reduces high blood pressure, cardiovascular diseases, stroke, diabetes, breast and colon cancer and it also reduces falls and fractures in adult. Physical activity improves function and bone health and is a fundamental determinant of energy consumption and thus energy balance and weight control [2]. Some studies have shown that the culture of urban life, the type of jobs, such as monotonous jobs and inactivity, have created special conditions that expose the human resources of universities and higher education institutions to psychologically and physical complications [3]. As an example, the research results of one of the universities of medical sciences in the country showed that 46.7% of the employees had low physical activity, 23.4% had moderate physical activity and 29.9% had good physical activity and there was a relationship between physical activity and the type of their jobs [4]. The results of another research on working women in Hamedan University of Medical Sciences showed that 65% of them did not exercise enough, 25.7% exercised irregularly and 8.7% exercised daily and regularly [5]. Social interaction is one of the key features of social media, which is considered one of the modern and non-present methods of learning, and their use has increased in the field of health promotion [6]. Iranian users rank fifth in the use of social media. Some of the advantages of using social media include increasing the access of people regardless of their age, education, race, and place of residence, and it also increases the self-confidence and sense of ownership of people [6]. WhatsApp is a social media messaging service for mobile phone. WhatsApp users consider the security of this program, the settings related to the user’s status, the right to choose to download or delete received files, the ability to search in conversations and sent texts and the ability to send messages to a large number of audiences as its most important features [7]. Health messages are designed to change the behavior of people in the health field. In campaigns, according to the audience and topics, three categories of messages are designed including informative, educational and motivational messages [8]. Public health planners often use motivational messages that play an important role in motivating people to promote and improve their health behavior, as a strategy to motivate people to adopt healthy behaviors or change unhealthy behaviors [9]. Prospect theory is one of the theories about decision making under uncertain conditions. Kahneman and Tversky have shown that people avoid of risk when they are in a situation of losses, but in a situation with a range of profits and benefits, they seek risk to some extent. According to the message framing theory, framing or presenting completely similar information about decision-making under conditions of uncertainty can be in one of the following two ways: potential benefits or potential losses. Framing of information can affect how people encode health related information. People respond differently to information framed in the form of gains and losses [10]. The positive frame of the message or gain frame messages focuses on the benefits of performing the behavior and Certain consequences, while the negative frame or loss frame messages focus on the health costs caused by not performing the behavior [11]. Salovey and Rothman state that message framing can be used to influence health decisions and motivate behavior change and also presented two hypotheses in the field of message framing: first, loss frame messages are more effective for people who engage in behaviors related to screening or early diagnosis, and second, gain frame messages for people who engage in preventive behaviors and with Certain consequences are involved and can be more effective [10]. After several decades of research on message framing and its effect, a definitive answer has not yet been given to the question of whether messages that emphasize the advantages of performing the behavior or the positive results of the behavior are more effective in encouraging people to perform the desired behavior or the message those who emphasize the consequences or negative results of not doing the behavior [12]. The results of the research indicate that the physical activity status of female university employees is not in favorable conditions and needs to improve behavior. Therefore, this study is designed to examine the physical activity status of female employees of universities and higher education institutions in Ahvaz city and to improve their physical activity behavior with an educational intervention based on message framing.

## Methods

### Study design and setting

This study was taken from the research plan approved with the code of ethics IR.ACECR.AVICENNA.REC.1399.028. This research a three-group intervention study, including a control group and two case groups. The number of samples was used to calculate the sample size in the Pocock analytical studies. The sample size calculation was based on similar study [13], and with 95% confidence, 90% statical power and 10% error, the sample size was calculated as 26 subjects (26 in each group). According to the probability of data loss 20%, finally the study started with 96 subjects (32 in each group). According to the research conducted, there are 13 universities and higher education institutions in Ahvaz city, this city is the capital of Khuzestan province, and it is a tropical region in the south of Iran. among them, based on random cluster sampling, three universities and higher education institutions were selected include Shahid Chamran University of Ahvaz (It is one of the largest governmental universities in Ahvaz), Payam Noor University of Ahvaz (It is one of the large governmental universities that trained virtual education) and ACECR- Khuzestan higher education institutions (It is one of the largest and private higher education institutions in Ahvaz). Then, based on a simple random sampling among these three universities and institutions was selected, Shahid Chamran University as the case group receiving loss framing messages, higher education institute of ACECR-Khuzestan as the case group receiving gain framing messages, and Payam Noor University as the control group. 32 female employees were selected based on available sampling and inclusion criteria, inclusion criteria to the research, not having a medical prohibition to do physical activity, having a mobile phone and being able to use it, access to the Internet, being able to use a computer, and not being pregnant and exclusion criteria was Inaccessibility, do not participate in education for more than 2 sessions and Reluctance to continue to the plan.

### Data collection tools

The data collection tool was 2 questionnaires, including a researcher-made questionnaire based on the message framing model and the International Physical Activity Questionnaire (IPAQ), which estimated women’s physical activity in the last week in terms of MET-minutes/week. This questionnaire contains 27 items and its interpretation and scoring were done based on the IPAQ scoring protocol [14]. The validity and reliability of the researcher-made questionnaire included Cronbach’s alpha 0.89, CVR 0.88, and CVI 0.89. Before conducting the study, sufficient information was given to the participants and written consent was obtained from them to participate in the study. Educational messages designed in two gain and loss frameworks regarding physical activity were sent to case groups in using mobile phone (Whatsapp) for 4 weeks and one day in between. In this study, one case group was sent messages about the benefits and advantages of doing physical activity or gain messages, and another case group was sent messages about the consequences of not doing physical activity or loss messages. In gain messages, sentences such as “strength of bones and prevention of osteoporosis, especially in women, is the result of regular physical activity”, in loss messages, sentences such as " inactivity causes loss of bone minerals, decrease bone mass and their weakening” was written. Between the end of the educational intervention and up to two months after that, an educational message was sent to the two case groups as a reminder every week. In order to determine the validity of the educational messages, the first version of the messages designed and reviewed by 10 experts in this field was. Data collection was done using questionnaires from all three studied groups before, immediately and 2 months after the intervention, by interview and self-report method. A specific code was given to each questionnaire, which was mentioned above. Code A was related to before the intervention, code B was related to immediately after the intervention and code C was related to 2 months after the intervention. Also, for the gain message group, the letter G, which abbreviation for GAIN, the loss message group, the letter L, which abbreviation for LOSS, and the control group, the letter C, which abbreviation for CONTROL, were given. For example, the questionnaire of the pre-test of the gain message group was coded as A-G-1. Immediately and 2 months after the end of the education, the questionnaires were completed again by all three groups.

### Data analysis

Data were analyzed using SPSS software version 26. Descriptive statistics such as mean ± Standard Deviation (SD), frequency and percentage for (qualitative variables) were used to describe the quantitative and qualitative variables. one-way analysis of variance, Pearson’s correlation coefficient, Repeated Measure, Paired t-Test was used to compare quantitative variables in each group. An Independent t-test was applied to compare the mean between the two groups, and a Chi-square test was used to compare the frequency of variables between the two groups. All tests were performed at a level of confidence of 95% and the data were analyzed by SPSS version 26 software at a significance level of 0.05 with s.

## Results

In this research, using the Kolmgrove-Smirnov (KS) statistical test, all data were normal and 2 subjects were excluded from each study group and the study continued with 90 women employees with an average age of 38.4 and a standard deviation of 5.3 participated. Using chi-square tests and one-way analysis of variance, no significant statistical difference (*P* < 0.05) was observed in the demographic variables between the case and control groups, and the groups were the same in this regard. The information related to demographic and general characteristics of the studied groups is given in tables number 1. The findings of the research showed that 5.5% of the studied subjects had a diploma or post-diploma, 94.4% had a bachelor’s degree and above, and most of them were married (77.9%). In terms of the number of children, 31 person (34.4%) have no children, 30 person (33.3%) have one child, 22 person (24.4%) have 2 children, 6 person (6.7%) have 3 children and 1 person (1.1%) had 4 children. Table number 2 compares the average score of physical activity behavior in the studied groups before, immediately and 2 months after the educational intervention. This table shows that the average score of physical activity behavior in the studied groups before and immediately after the educational intervention has no significant statistical difference, but two months after the educational intervention, the difference between the groups has become significant. Based on the repeated measure test, the significant difference observed in the group receiving gain messages was due to the significant difference in the stages before and immediately after the intervention, before and 2 months after the intervention, and immediately and 2 months after the intervention. But in the group receiving the loss message, this difference was observed only in the stages before and 2 months after the intervention and immediately and 2 months after the intervention, and no difference was observed in the stage before and immediately after the intervention.

**Table 1:** Comparison of the demographic characteristics of the studied subjects

	Variable		Gain frame message	Loss frame message	Control	*p*-value
Age	Years	37.2 ± 4.7	39.5 ± 6.6	38.4 ± 4.2	0/23	
Children (%)	0	10(33.3)	13(43.3)	8(26.7)	0.09	
	1	13(43.3)	9(30)	8(26.7)		
	2	7(23.3)	7(23.3)	8(26.7)		
	3	0	1(3.3)	5(16.7)		
	4	0	0	1(3.3)		
Level of education (%)	Bachelor’s degree and higher	29(96.7)	26(86.7)	30(100)	0.06	
	Diploma and Associate’s Degree	1(3.3)	4(13.3)	0(0)		
Marital status	Single	5(16.7)	7(23.3)	7(23.3)	0.76	
	Married	25(83.3)	23(76.7)	23(76.7)		

**Table 2:** Comparison of the mean and standard deviation of physical activity behavior in the studied groups before and after the educational intervention

Physical activity behavior	Before intervention Mean ± SD	Immediately after the intervention Mean ± SD	2 months after the intervention Mean ± SD	*p*-value Repeated Measure
Gain frame message	5436.66 ± 3950.05	6403.37 ± 3481.46	8338.63 ± 4395.07	0.001
Loss frame message	5583.91 ± 3922.25	5947.95 ± 3598.06	6415.10 ± 3909.48	0.006
Control	5368.8 ± 2767.79	5219.3 ± 3055.23	5049.93 ± 2538.27	0.8
*p*-value One-way ANOVA	0.97	0.39	0.004	

In table number 3, subjects with physical activity less than 600, 600 to 3000 and more than 3000 according to MET are placed in the group with light, moderate and intense physical activity, respectively. the type of physical activity (light-moderate-intense) in the studied groups has been compared before and after the educational intervention, the chi-square statistical test showed the type of physical activity before and immediately after the intervention between the groups has no significant difference. But was observed a significant difference 2 months after the intervention.

**Table 3:** The type of physical activity among the studied groups intervention

Group Time	Gain frame message (%)			Loss frame message (%)			Control (%)			*p*-value
Before intervention	Light	Moderate	Strenuous	Light	Moderate	Strenuous	Light	Moderate	Strenuous	0.7
	0	8(26.7)	22(73.3)	0	6(20)	24(80)	0	6(20)	24(80)	
Immediately after the intervention	0	2(6.6)	28(93.4)	0	5(16.7)	25(83.3)	1(3.3)	7(23.3)	22(73.3)	0.12
2 months after the intervention	0	1(3.3)	29(96.7)	0	4(13.3)	26(86.6)	1(3.3)	6(20)	23(76.7)	0.006

Table number 4 shows that the difference in the average score of physical activity behavior between the control and gain messages groups and also between the groups of loss messages and gain messages is statistically significant. According to IPAQ, the higher the score of the questionnaire, the more physical activity has been performed and by comparing the increase of this score in the intervention groups, 53% improvement in physical activity is observed in the gain message group and 15% in loss massage group, while no significant change is observed in the control group. Using the LSD post hoc test, it can be understood that the effect of gain and loss messages on physical activity behavior is different and it seems that gain messages have more impact than loss messages.

**Table 4:** Comparison of the amount of physical activity of two groups after the intervention using LSD post-test

physical activity	Pair wise comparison of groups		*p*-value
Before intervention	Gain frame message	Loss frame message	0.8
		Control	0.9
	Loss frame message	Gain frame message	0.8
		Control	0.8
	Control	Gain frame message	0.9
		Loss frame message	0.8
Immediately after the intervention	Gain frame message	Loss frame message	0.6
		Control	0.1
	Loss frame message	Gain frame message	0.6
		Control	0.4
	Control	Gain frame message	0.1
		Loss frame message	0.4
2 months after the intervention	Gain frame message	Loss frame message	0.04
		Control	0.001
	Loss frame message	Gain frame message	0.04
		Control	0.1
	Control	Gain frame message	0.001
		Loss frame message	0.1

## Discussion

According to the results of this study, the studied groups, including the groups that received gain messages, loss messages, and control groups, did not have any difference in physical activity behavior before intervention. Also, immediately after educational intervention, there was no difference in the physical activity behavior of the groups, but two months after the educational intervention, a significant difference was observed between the three groups. Physical activity behavior in the intervention groups increased significantly after receiving the messages compared to before it, and it shows that education based on message framing using mobile phones (What Sapp) improved physical activity behavior. Alshahrani et al. [15] showed in a study that the educational intervention based on social network and What Sapp messenger caused a significant improvement in the physical activity of the studied students. Also, Baji et al. [16] in research that investigated the effect of gain and loss messages through mobile phone text messages on foot self-care behavior in women with type 2 diabetes showed that the presentation of messages in both framings caused a significant increase in foot self-care behavior in the case groups compared to the control group. The findings of Noroozi et al.‘s study [17] also showed that gain and loss framing increase the awareness, self-efficacy and self-management of diabetic patients. ghajari et al. [9] also showed that both gain and loss framing increase positive attitude and consumption of calcium-rich foods in students. One-way analysis of variance with LSD post hoc test showed that the mean score of physical activity behavior between the control and gain message groups in the stage immediately after the intervention and two months later and between the loss message and gain message groups had a statistically significant difference. In fact, the effect of gain and loss messages on physical activity behavior is different, and gain messages have been more effective than loss messages. the results of these studies and so studies Parrott et al. 18), Gallagher et al. [18], Latimer et al. [19] showed that the group that received messages with a positive frame reported more physical activity than the group that received loss messages, and this finding is in agreement with the results of this study. While the findings of other studies such as the results of Baji et al. [20] and Bong et al. [21] showed that loss messages were more effective than gain messages in persuading diabetic patients to perform physical activity behavior. In Gilbert et al.‘s study [22], both gain and loss message frames were effective in increasing the motivation of physical activity in students, but there was no statistically significant difference between the two types of messages. Perhaps this discrepancy is due to the different educational methods and content and the characteristics of the studied subjects. In the current study, physical activity was divided into three types: light, moderate, and intense. After the educational intervention, the physical activity of the case groups was improved from light to intense compared to the control group, so that all people in the gain message group improved from moderate to intense physical activity in the two-month phase after the educational intervention. In a study conducted by Hazavehei [23] on students, by following the study, the type of physical activity among the studied students improved and they reached the stage of intense physical activity. Also, in a study conducted by Solhi et al. [24] on students in order to improve physical activity behavior, the amount of physical activity among the students of the intervention group increased significantly and some of them also engaged in intense physical activity. These results were agreement with the findings of the present study. But, in the study of Eskandari et al. [25], the type of physical activity in the intervention group was increased from light to moderate, but none of the subjects had reached the stage of intense physical activity. Also, the findings study of Pirasteh et al.‘s [26] in the field of improving the physical activity of the studied mothers showed that the majority of the mothers’ activities before the educational intervention were light activities and sedentary and were in the form of sitting activities and little time spend on sports activities and after the intervention, although their physical activity increased, none of them had reached an intense level. These findings were not agreement with the findings of the present study and may be due to the research samples and the method of conducting the study. According to studies the positive frame of the message or gain frame messages focuses on the benefits of performing the behavior and Certain consequences [11, 27] Considering that physical activity has a Certain consequences and certain benefits that can prevent many chronic diseases, it seems that according to this research, gain frame messages should be more effective than loss frame messages.

## Conclusion

The present study was designed and conducted in order to survey the effect of education based on the framing of educational messages through mobile phone (WhatsApp) on the physical activity improve of female employees of universities and higher education institutions in Ahvaz. The results obtained from this study showed that education based on the framing of educational messages through social networks (WhatsApp) in order to improve physical activity behavior in women can be effective. Also, regarding physical activity behavior, it seems that educational messages with a positive frame or gain messages can be more effective than loss messages.

## Limitations

This study has some limitations. Firstly, the study was conducted on female employees, and its results can only be generalized to women, and it is suggested to conduct it on male employees as well. Secondly, the training method was only to send messages, and because the work of female employees is a lot, such messages may have been given little attention. Studies are needed to be conducted among female employees in other countries in the future.

## Data Availability

The datasets used and/or analyzed during the current study are available from the corresponding author on reasonable request.
